# Reducing Children's Media Viewing

**DOI:** 10.1155/2011/287243

**Published:** 2011-12-14

**Authors:** Erin Clyne, Leonard A. Jason

**Affiliations:** Center for Community Research, DePaul University, 990 W. Fullerton Avenue, Chicago, IL 60614, USA

## Abstract

This case study evaluates the use of a Restrictive aide and the complementary use of parental mediation techniques to reduce media viewing among children. A family was provided a TV Token Timer, which involved a positive reward in exchange for participating in activities outside of watching television. Media viewing habits decreased from the baseline to the completion of the intervention at week 8 for both children. The parent reported high levels of Instructive mediation and positively evaluated the family's use of the TV Token Timer.

## 1. Reducing Children's Media Viewing

Media use has been increasing among youth in the United States due to advances in technology and availability of mobile devices [1]. The average American 8–18 years old spends over 7.5 hours watching or interacting with media on an average day, but this does not include school work or cell phone talk time/texting. Rates differ depending on the child's age, ethnicity, gender, and so forth. Rules and expectations set by parents also affect overall media exposure.

Bybee et al. [2] found that the type of media guidance utilized was partially based on the parent's perceived social effects of television, gender of the parent, and the child's age. Valkenburg et al. [3] found that parents used three mediation techniques: Restrictive, Instructive, and Social Coviewing. Restrictive mediation involves the parent setting specific viewing times and which programs are suitable. Instructive mediation requires the parent to explain the motivation of a character, reality of the situation, or what character behaviors are acceptable. The technique that requires the least time and effort on behalf of the parent is Social Coviewing, because the interaction between parent and child does not necessarily provide an explicit approval or disapproval by the parent (i.e., watching the program with little or no discussion).

Researchers at DePaul University have tracked changes in media consumption in the home by children in previous studies using a Token Timer as a Restrictive mediation aide and have found that it resulted in a reduction of electronic media viewing [4]. The current study focuses on how use of the Token Timer affects electronic media consumption in a single household, and how it might be related to the use of the three parent mediation techniques described by Valkenburg et al. [3].

## 2. Method

### 2.1. Participants

A family was recruited, and it included one male child aged 13 (Child 1) and one female child aged 11 (Child 2). The parent who provided data was the married Caucasian mother who had a college degree and worked full time as a school teacher.

### 2.2. Procedure

Behavioral technologies to assist parents in reducing their children's media use were previously described by Jason and Hanaway [5]. These behavioral technologies involved a child having to earn a token based on an agreement with the parent(s) regarding participation in academic, physical, or social activities. The token could later be inserted into a Token Timer [6] for a viewing time of 35 minutes on a single electronic device. The Token Timer is an electrical device that connects to a TV by way of a power cord to act as an intermediary output. In our case study, the Token Timer was attached to the television, which also mediated access to watching the VCR/DVD player. Tokens are then inserted into the Token Timer to activate the device for 35 minutes per token.

The procedure was comprehensively described elsewhere [4]. The consent of the parent and assent for each child were attained prior to the start of the study. The mother was also asked to complete the Television Mediation Scale (TMS) questionnaire [3] at the beginning and end of the study, along with the Customer Satisfaction survey only at the completion of the study. Daily media viewing of the children was collected by the parent for the duration of the seven-week intervention.

## 3. Materials

### 3.1. Television Mediation Scale

The TMS consists of 15 items which ask how the parent monitors the child's TV viewing [3]. There are five questions for each of the three mediation methods (Restrictive, Instructive, and Social Coviewing). Each question item was rated with a “Never,” “Rarely,” “Sometimes,” or “Often” on a scale of one to four, with “Never” equating to one. The individual items were then grouped by mediation method, and the mean (*M*) and standard deviation (SD) for each mediation method were calculated. The *M* ranges from one to four, with higher values indicating more frequent use of the particular mediation method.

### 3.2. Media Form

This form allowed the parent to record the number of hours each child spent daily with television programs, VCR/DVD, video games, computer games, internet sites, movies at the theater, and portable device usage. Data were collected from week one through week eight. The types of media were categorized into three separate groups. The first group included television and VCR/DVD use because it was attached to the Token Timer. The second group included video games, computer games, internet sites, and movies at the theater. The third group was portable device use, and there was less parental control regarding use by the children.

### 3.3. Customer Satisfaction Questionnaire

This questionnaire asked the parent about his/her opinion about any possible changes that occurred in the children during the two months that the device was utilized.

## 4. Results

### 4.1. Television Mediation Scale

When the mother rated her use of mediation measures at week one, she indicated more frequent use of Instructive mediation (*M* = 4.00, SD = 0.00), followed by Restrictive (*M* = 3.60, SD = 0.55) and Social Coviewing (*M* = 3.4, SD = 0.89). At postintervention, Instructive mediation was the preferred method (*M* = 4.00, SD = 0.00), with some decrease in frequency among both Restrictive (*M* = 3.00, SD = 1.00) and Social Coviewing (*M* = 2.80, SD = 0.45) mediation. We did not assess how these methods were utilized differently between children.

### 4.2. Media Form

**Figure 1:**
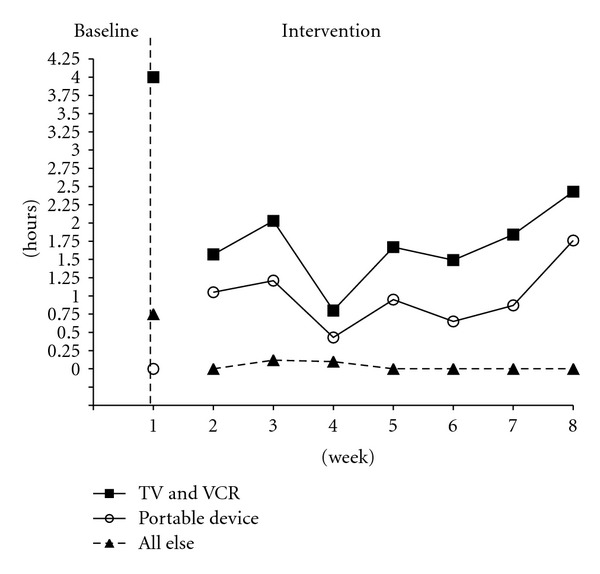
Child 1 averaged weekly media use by form over time.

Figure 1 shows data for Child 1 over time with a total decrease in electronic media viewing time of just over 30 minutes (baseline = 4.75 hours, week 8 = 4.19 hours). One-week averages resulted in a drop in TV viewing from 3 hours to 2.14 hours, DVD viewing dropped from 1 hour to 0.29 hours, both Videogame (baseline = 0.25 hours) and Internet use (baseline = 0.75 hours) decreased to zero hours, computer and movie theater attendance remained at zero hours, and portable device use increased from zero hours to 1.76 hours.

**Figure 2:**
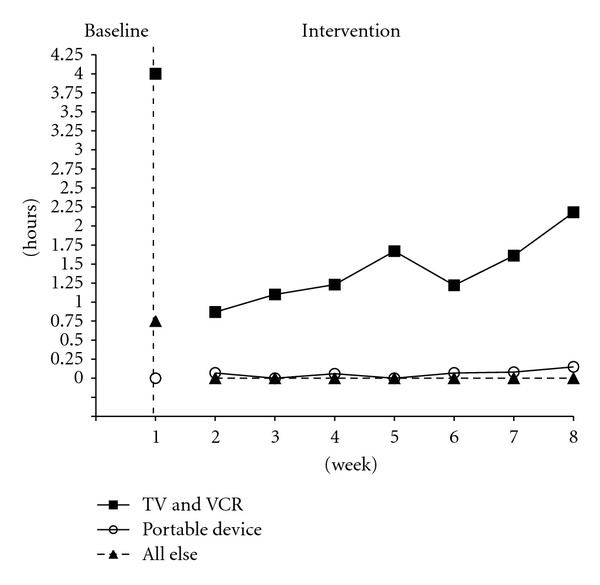
Child 2 averaged weekly media use by form over time.

Child 2 averaged a decrease of just less than two and a half hours (baseline = 4.75 hours, week 8 = 2.33 hours) (see Figure 2). Computer usage and movie theater attendance remained at zero hours. TV viewing was reduced from 3 hours to 1.89 hours, DVD viewing was reduced from 1 hour to 0.29 hours, video gaming went from less than 0.25 hours to zero, and internet use went from 0.50 hours to zero. Portable device use was the only media form that increased from zero to 0.15 hours a day on average.

### 4.3. Customer Satisfaction Questionnaire

Upon completion of the study, the mother completed the questionnaire and indicated that there was an overall positive response within her family with the results of the Token system, “…All in all I feel like our household runs as more of a group effort today because of the intervention of the Token Timer.” The only negative response was in regard to Child 1′s increased use of portable devices.

## 5. Discussion

The mother indicated that Instructive mediation was used most frequently at the baseline and after intervention indicating her preference throughout the study for this mediation method. This finding contrasts with both Valkenburg et al.'s [3] and Bybee et al.'s [2] results, which indicated that Social Coviewing is often the most utilized approach. The current case study found high levels of time and involvement of the parent with the focus on Instructive mediation of children, compared to Bybee et al.'s [2] study, and these differences may be due to the mother's occupation which involves teaching children on a daily basis.

According to the records of media usage, all electronic media forms tracked decreased over time except for portable devices. The parent might have had less control over portable device use, as it was not attached to the Token Timer. Child 2 had greater decreases in electronic media use, and this child was younger whereas Child 1 was older and part of an age group when peers become more influential, thereby diminishing parental influence [7]. It should be noted that, after an initial reduction, trends in media use increased for both children. Jason [8] found that when the contingencies were removed, the prosocial behaviors were maintained.

One limitation to this study is that it is unclear what the longer-term influences of the Token system might be with the children. Another limitation is that sometimes the children found creative ways to overcome the Token system. For example, the parent reported that Child 1 influenced Child 2 to bypass the Token system by manipulating the Token Timer to accept pennies as a substitute. The parent assured the investigators that media use continued to be tracked during this time, and once the parent discouraged the manipulation of the Token Timer, the children were no longer able to use pennies. The number of hours utilized for each media form was not individually differentiated between the two children at week one; therefore, caution is urged when analyzing changes between week one and the intervention.

Despite these limitations, the family was satisfied with the program overall which reflects previous media reduction studies through DePaul University [4, 8, 9]. Future research should be conducted with larger samples and include follow-up periods. It is also important to continue assessing what occurs to different types of media viewing when separate TV and VCR/DVD viewing device reductions are targeted.
